# *Escherichia coli* B2 strains prevalent in inflammatory bowel disease patients have distinct metabolic capabilities that enable colonization of intestinal mucosa

**DOI:** 10.1186/s12918-018-0587-5

**Published:** 2018-06-11

**Authors:** Xin Fang, Jonathan M. Monk, Nathan Mih, Bin Du, Anand V. Sastry, Erol Kavvas, Yara Seif, Larry Smarr, Bernhard O. Palsson

**Affiliations:** 1 Department of Bioengineering, University of California San Diego, 9500 Gilman Drive, La Jolla, 92093 CA USA; 2 Department of Bioinformatics and Systems Biology, University of California San Diego, 9500 Gilman Drive, La Jolla, 92093 CA USA; 3 Department of Computer Science and Engineering, University of California San Diego, 9500 Gilman Drive, La Jolla, 92093 CA USA; 4 California Institute for Telecommunications and Information Technology, University of California San Diego, 9500 Gilman Drive, La Jolla, 92093 CA USA; 5 The Novo Nordisk Foundation Center for Biosustainability, Technical University of Denmark, Anker Engelunds Vej 1 Bygning 101A, 2800 Kgs., Lyngby, Denmark; 6 Department of Pediatrics, University of California San Diego, 9500 Gilman Drive, La Jolla, 92093 CA USA

**Keywords:** Metabolic modeling, Pan-genome analysis, Inflammatory bowel disease

## Abstract

**Background:**

*Escherichia coli* is considered a leading bacterial trigger of inflammatory bowel disease (IBD). *E. coli* isolates from IBD patients primarily belong to phylogroup B2. Previous studies have focused on broad comparative genomic analysis of *E. coli* B2 isolates, and identified virulence factors that allow B2 strains to reside within human intestinal mucosa. Metabolic capabilities of *E. coli* strains have been shown to be related to their colonization site, but remain unexplored in IBD-associated strains.

**Results:**

In this study, we utilized pan-genome analysis and genome-scale models (GEMs) of metabolism to study metabolic capabilities of IBD-associated *E. coli* B2 strains. The study yielded three results: i) Pan-genome analysis of 110 *E. coli* strains (including 53 isolates from IBD studies) revealed discriminating metabolic genes between B2 strains and other strains; ii) Both comparative genomic analysis and GEMs suggested that B2 strains have an advantage in degrading and utilizing sugars derived from mucus glycan, and iii) GEMs revealed distinct metabolic features in B2 strains that potentially allow them to utilize energy more efficiently. For example, B2 strains lack the enzymes to degrade amadori products, but instead rely on neighboring bacteria to convert these substrates into a more readily usable and potentially less sought after product.

**Conclusions:**

Taken together, these results suggest that the metabolic capabilities of B2 strains vary significantly from those of other strains, enabling B2 strains to colonize intestinal mucosa.The results from this study motivate a broad experimental assessment of the nutritional effects on *E. coli* B2 pathophysiology in IBD patients.

**Electronic supplementary material:**

The online version of this article (10.1186/s12918-018-0587-5) contains supplementary material, which is available to authorized users.

## Background

Alteration of the composition of the gut microbial community has been implicated in inflammatory bowel disease (IBD) [1]. Several studies have shown that the abundance of *E. coli* in the gut microbiome of IBD patients is higher compared to healthy subjects [1–3]. In comparison with healthy controls, *E. coli* isolates from IBD patients mainly belong to B2 and D phylogroups, including extraintestinal pathogenic *E. coli* strains (ExPEC) [4]. In particular, a specific *E. coli* pathotype, adherent-invasive *E. coli* (AIEC), has been shown to be a leading bacterial trigger of IBD [5]. AIEC strains mostly belong to B2 phylogroups [3]. They are able to adhere to intestinal epithelial cells and survive and replicate within macrophages, yet the specific genetic determinants of this pathotype are still unknown [6].

In recent years, several comparative studies were performed on *E. coli* isolates to understand their pathogenicity in IBD [6–8]. Additionally, a few specific *E. coli* strains associated with IBD have been characterized in detail, including LF82 [9], UM146 [10], and NRG857c [11], all of which are in phylogroup B2. Most of these studies have focused on comparative phenotypic assays and genome analysis such as virulence factor determination. A previous study has shown that strains in B2 phylogroup possess certain virulence factors including adherence genes, that allow them to persist within the human intestine, while strains in A and B1 phylogroups are primarily transient *E. coli* strains [12]. However, the systems biology of IBD-related *E. coli* strains, such as metabolic network reconstructions that elucidate nutrient niches, remains unexplored.

Genome-scale models (GEMs) represent a mathematical framework that enables a mechanistic description of metabolic functions and how they relate to physiological properties. GEMs have been used extensively to contextualize multi-omics data as well as to understand the genetic basis of phenotypic functions [13–16]. The metabolism of *E. coli* strains has been studied extensively, enabling the development of GEMs for a wide range of *E. coli* strains. Recent studies have shown that strain-specific GEMs are necessary to capture the variation in metabolic capabilities in different strains [17], as the *E. coli* pan-genome is estimated to have more than 45,000 genes [18].

In this study, we analyzed the metabolic capabilities of B2 *E. coli* strains prevalent in IBD patients using pan-genome analysis and genome-scale metabolic models. We look at a large set of *E. coli* strains from IBD patients and healthy controls, as well as strains from other origins, to see if we could identify any common metabolic patterns associated with IBD pathophysiology in B2 strains. We showed that specific metabolic capabilities of the B2 group allow them to colonize intestinal mucus and become resident *E. coli* strains in the human gut.

## Results

### Strain collection studied

We collected available genomes of *E. coli* isolates from previous IBD studies - 53 *E. coli* strains (22 AIEC, 31 non-AIEC), most of which were isolated from intestinal biopsies of both IBD patients and healthy subjects (see Additional file 1: Table S1). 52 of the 53 strains belong to B2 groups; however these studies did not include many genome sequences in other phylogroups from healthy controls [6]. Thus, we set out to compare these isolates with 57 other *E. coli* strains including commensal strains and those that exhibit extra-intestinal and intra-intestinal (InPEC) pathotypes. Of the 57 other *E. coli* strains, 14 strains belong to phylogroup B2, and the other strains span various phylogroups (see Additional file 2: Figure S1).

### Strains in B2 phylogroup contain distinct metabolic genes compared to strains in other phylogroups

To identify important metabolic features in B2 *E. coli* strains, we first constructed the pan-genome from the 110 strains, including 53 strains isolated from both IBD patients and healthy controls. A pan-genome for the 110 strains was built using CD-HIT [19] with 80% similarity setting (see “Methods” section). We found an open pan genome with 16,091 orthologous genes (see Additional file 2: Figure S2), among which 2979 are metabolic genes annotated by Enzyme Commission (EC) numbers. Out of all the metabolic genes identified, only 1081 clusters are conserved across 110 strains. We then further investigated the distribution of the 1898 accessory metabolic genes in 110 strains.

**Fig. 1:**
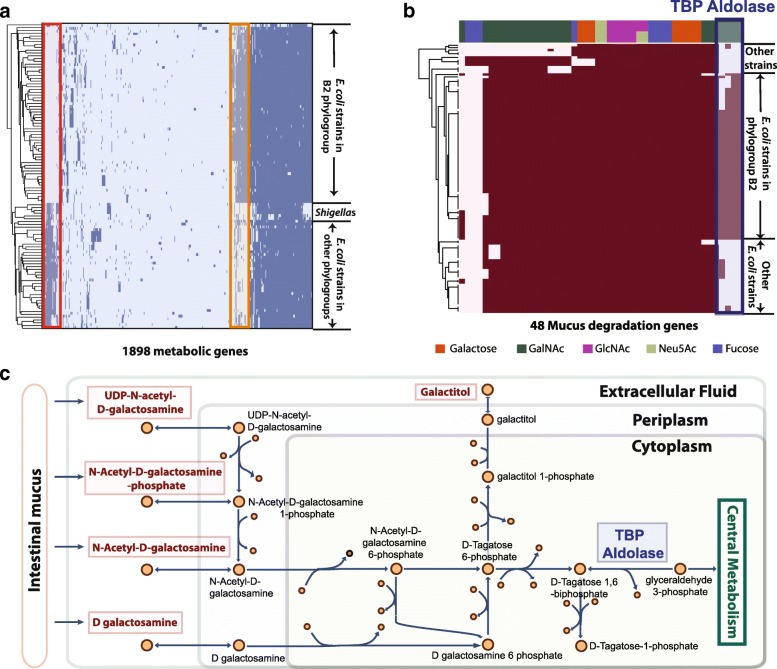
Pan-genome analysis shows B2 strains contain distinct metabolic genes. **a** 110 strains are clustered by the presence/absence of 1898 accessory metabolic genes. Genes in the red box are primarily absent from B2 strains, while genes in the orange box are more prevalent in B2 strains. **b** Presence and absence of genes involved in mucus degradation in 110 *E. coli* strains (genes are colored based on their functions in monosaccharides degradation). The four highlighted genes annotated as tagatose bisphosphate (TBP) aldolase are more prevalent in B2 strains. **c** Metabolic pathways of degradation of five nutrient sources involve TBP aldolase

We found that most B2 strains have distinct metabolic genes compared to strains in other phylogroups (Fig. 1a). Metabolic genes highlighted in the red box in Fig. 1a are missing from most B2 strains, while genes highlighted by the orange box are more prevalent in B2 strains (present in < 15% non-B2 strains and > 80% B2 strains). We then selected the 100 most differentiating metabolic genes between B2 strains and strains in other phylogroups using the SelectKBest function from scikit-learn package [20] (see “Methods” section). Of the selected genes, 53 genes are more prevalent in B2 strains and encode various functions including energy production, amino acid metabolism, carbohydrate metabolism, and metal binding. GO enrichment analysis [21] suggested that these genes are only enriched for tricarboxylic acid (TCA) cycle (False discovery rate (FDR) adjusted *p*-value =3.89×10^−2^). Upon further investigation, we found that B2 strains possess an extra set of *sucABCD* genes that share ∼ 50% sequence identity with the original *sucABCD* genes present in all strains. These four genes encode the important enzymes in the TCA cycle: alpha-ketoglutarate dehydrogenase (*sucAB*) and succinyl coenzyme A synthetase (*sucCD*) [22]. Experiments are needed to characterize the function and importance of these gene variants in B2 strains. The remaining 47 metabolic genes that are primarily absent from B2 strains are enriched for folic acid catabolism (FDR adjusted *p*-value =4.52×10^−2^), 3-phenylpropionate catabolism (FDR adjusted *p*-value =7.65×10^−4^) and putrescine catabolism (FDR adjusted *p*-value =1.97×10^−3^). To explore the relationship between the metabolic functions and nutrient niches, we further investigated specific metabolic genes.

### IBD isolates and other ExPEC strains in the B2 phylogroup contain unique metabolic genes that enable them to utilize mucus glycan

We focused on elucidating the metabolic genes that allow *E. coli* strains of the B2 group to reside within intestinal mucosa. Glycans of the intestinal mucus can be utilized as a source of carbon and energy by intestinal microbiota, and depletion of mucus is associated with Crohn’s disease [23]. Research has shown that commensals are mostly involved in cleavage of glycans into monosaccharides, while pathogens such as *E. coli* utilize the five monosaccharides released by commensals: fucose, galactose, N-acetylgalactosamine (GalNAc), N-acetylglucosamine (GlcNAc), and N-acetylneuraminic acid (Neu5Ac) [24]. Therefore, we performed comparative analysis on 48 genes (see Additional file 1: Table S2) involved in mucus degradation among the 110 strains. These genes were identified from a previous study on degradation of mucin glycans [24] (see “Methods” section). The resulting heatmap (Fig. 1b) illustrated that although many genes have similar distribution among all 110 strains, four genes that are involved in tagatose 1,6-bisphosphate (TBP) aldolase are more prevalent in the B2 phylogroup (highlighted in Fig. 1b). These genes are also present in a few D strains (see Additional file 2: Figure S3), which was expected since both B2 and D strains are commonly found in IBD patients [4]. TBP aldolase converts TBP to dihydroxyacetone phosphate and glyceraldehyde-3-phosphate that is subsequently fed into central metabolism (Fig. 1c). Two of the four genes are identified to be variants of known TBP aldolase subunit GatY, while the other two genes are annotated to be TBP aldolase and related Type B Class II aldolases, but have not been well-characterized.

We then performed structural analysis to confirm the substrates and functions of the four genes annotated as TBP aldolases. We obtained homology models for the four proteins and compared them against the crystallized structure of the known TBP aldolase [25] and fructose-1,6-bisphosphate (FBP) aldolase [26], since these two enzymes are highly similar. The models were found to be more structurally similar to the known TBP aldolase, rather than the FBP aldolase. This conclusion mainly arose due to an extended sequence of amino acids in FBP aldolase compared to TBP aldolase. This sequence extends the *α*10 loop - *α*11 arm [25] that results in the main differentiating feature between the enzymes’ monomer subunits. Additionally, differences in the substrate binding sites lead to steric restrictions in FBP aldolase that constrain its substrate to be highly specific for FBP. All four predicted TBP aldolases contain different sets of residues, suggesting that they have the potential to greatly alter these steric restrictions and allow a wider range of substrates (including TBP) to enter the binding site. These differences are outlined in Additional file 2.

The presence of these additional TBP aldolases potentially gives B2 strains an advantage to thrive in intestinal mucosa, as TBP aldolase is an important enzyme that is involved in the degradation of GalNAc and its derivatives [24], as well as galactitol (Fig. 1c). These B2 strains are likely to be more efficient in breaking down these nutrient sources produced from mucus glycan, thus having an advantage to survive in intestinal mucosa. Based on these observations of differentiating metabolic features, we next utilized genome-scale models to obtain a systems-level understanding of the metabolic capabilities of B2 and other strains.

### Reconstruction of draft genome-scale metabolic models for 110 strains

**Fig. 2:**
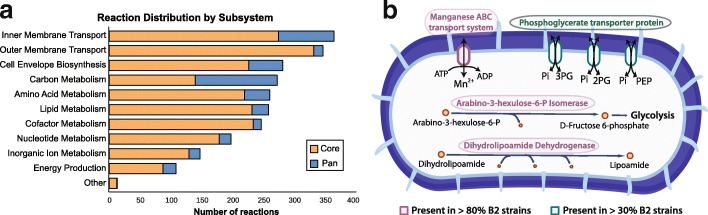
Reactions distribution in 110 GEMs. **a** Distribution of pan and core reactions in different systems for 110 *E. coli* models. **b** Unique reactions in models of B2 strains. Reactions present in more than 80% and 30% of B2 models are shown

GEMs can be used to systematically determine the metabolic capabilities of a strain [14]. We built GEMs of the 110 strains by mapping their genomes to a pan-metabolic model that contains all the reactions and genes collected from a previous *E. coli* multi-strain study [17] (see “Methods” section). We first identified 2485 core metabolic reactions that are present in all 110 GEMs, and 441 accessory reactions that are absent from at least one GEM. Functional distribution of pan and core reactions indicates that most accessory reactions are involved in transport processes, carbon metabolism and cell envelope biosynthesis (Fig. 2a), suggesting that these strains are adapted to their own nutrient niches. Transporters in bacteria are adapted to their environment in order to best utilize the nutrients available [27]. Moreover, some accessory reactions in the category of cell envelope biosynthesis are involved in the synthesis of lipopolysaccharides (LPS), molecules also known as endotoxins, that contribute to the pathogenicity of *E. coli* strains. The toxic portion of LPS, lipid A, induces a release of host proinflammatory cytokines and causes infection within the host [28]. These models illustrate potential variation in LPS components, which could directly correlate with host inflammatory state in IBD patients.

We specifically examined the distribution of reactions in B2 and non-B2 strains. We investigated the 26 reactions that are unique to B2 strains, and identified three reactions that exist in more than 80% of B2 strains: manganese ATP-binding cassette (ABC) transporter, arabino-3-hexulose-6-phosphate isomerase, and reversible dihydrolipoamide dehydrogenase (Fig. 2b). Strains in both B2 and non-B2 groups are able to transport manganese via permease, while only B2 strains are able to transport manganese via ABC transporter. Knowledge of the other two enzymes is limited, and are thus potential experimental targets. In addition, three other transport reactions involved in the uptake of phosphoenolpyruvate, D-glycerate 2-phosphate, and D-glycerate 3-phosphate are also present in more than 30% of B2 strains. This is due to the presence of the *pgtP* gene, originally found in *Salmonella*, which is responsible for phosphoglycerate transport [29]. Thirteen reactions are missing from all B2 models, but were later found to be uncommon in non-B2 strains, as well. To further elucidate the differences in metabolic functions between B2 and non-B2 strains, we simulated growth of these strains on a variety of nutrient sources.

### Comparative analysis of GEMs highlights metabolic capabilities unique to B2 *E. coli* strains

**Fig. 3:**
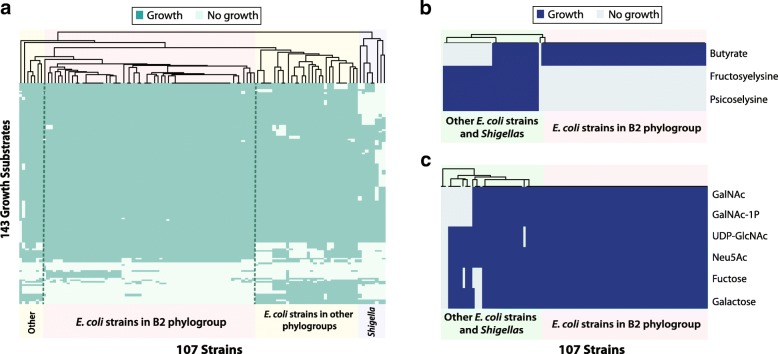
Simulated growth capabilities of 107 GEMs on various nutrient sources. **a** 107 strains are clustered by simulated growth capabilities on 143 differentiating nutrient sources. **b** Simulated growth on monosaccharides and their derivatives from mucus glycan. **c** Simulated growth on butyrate, fructoselysine and psicoselysine

Growth simulation of GEMs predicted that strains in the B2 group, including 52 isolates from IBD studies, share similar metabolic capabilities (Fig. 3a), regardless of the IBD status of their hosts. Growth simulations were performed for 649 substrates under aerobic conditions, as research has shown that aerobic respiration is required for *E. coli* to colonize the mouse intestine [30]. B2 strains isolated in IBD studies displayed distinct metabolic capabilities compared to other InPEC strains, including Enterotoxigenic *E. coli*, Enteropathogenic *E. coli*, and Enteroaggregative *E. coli*, but are similar to ExPEC strains in B2 groups such as Uropathogenic *E. coli* strains. This result is interesting since a subset of AIEC and other InPEC strains all colonize epithelial cells in the small intestine [1, 31] and thus likely share a preferred microenvironment, yet they display distinct metabolic capabilities. Specifically, B2 strains were predicted to be unable to grow on certain substrates, including psicoselysine, fructoselysine, meliobiose, cyanate, phenylpropanoate and L-Xylulose (Table 1).

**Table 1:** Growth substrates that differentiate *E. coli* strains in B2 phylogroup from other strains

Phylogroup B2				Other phylogroups		
Growth Substrates	AIEC IBD (%)	Commensal IBD (%)	ExPEC (%)	InPEC (%)	Commensal (%)	Shigella (%)
Fructoselysine	0	3.2	0	90.9	69.2	87.5
Psicoselysine	0	3.2	0	90.9	69.5	87.5
Melibiose	4.6	6.5	33.3	81.8	57.7	100
L-Xylulose	4.6	6.5	33.3	45.5	69.2	12.5
Cyanate	4.6	6.5	33.3	90.9	65.4	0
Phenylpropanoate	4.6	6.5	33.3	90.91	65.4	12.5
Xanthosine 5’-phosphate	77.3	93.6	77.8	45.5	38.5	0
Xanthosine	77.3	93.6	77.8	45.5	38.5	0

We then investigated the most differentiating nutrient sources between B2 and non-B2 strains: fructoselysine and psicosylysine, also known as amadori products, that are abundantly formed in heated food and decomposed by microorganisms in the large intestine [32]. Further investigation using GEMs suggested that both the fructoselysine transporter and *frl* operon, including fructoselysine 6-kinase and fructoselysine 6-phosphate deglycase, are missing from *E. coli* strains in phylogroup B2, resulting in their inability to metabolize fructoselysine and psicoselysine. This result is consistent with experimental data describing growth of mutant *E. coli* strains on fructoselysine [33]. It is possible that B2 *E. coli* strains do not use these substrates directly, but instead use their derivatives produced by other organisms. Research has shown that *Intestinimonas AF211* and related bacteria that are abundantly present in colonic samples are able to convert amadori products into butyrate [34], a substrate that could be metabolized by all *E. coli* strains in the B2 group, while 50% of the non-B2 strains failed to do so (Fig. 3b). This could potentially explain the lack of degradation enzymes for fructoselysine and psicoselysine in B2 strains: by dispensing these enzymes, B2 strains could rely on neighboring bacteria to convert these substrates into a more readily usable and potentially less sought after product. Additionally, butyrate plays an important role in maintaining intestinal homeostasis and has therapeutic potential for IBD patients [35]. The elevated abundance of B2 strains in IBD patients and their capability to metabolize butyrate could potentially be related to the decreased concentration of butyrate in feces of IBD patients [36] and inflammation.

Moreover, model simulations showed strains in phylogroup B2 differ from other strains in their ability to catabolize mucus monosaccharides. We examined the simulated growth capabilities of *E. coli* strains on five monosaccharides and their derivatives that are released from intestinal mucus glycan by commensals. Simulated growth results suggest that 100% of the B2 strains can utilize all tested monosaccharides as their sole carbon source, while only 65% of the strains from other phylogroups can utilize all six substrates tested (Fig. 3c).

## Discussion

In this study, we delineated the specific metabolic capabilities of *E. coli* B2 strains that are found to be prevalent in IBD patients. Our study used pan-genome analysis of metabolic genes and the growth capabilities they confer. The study yielded three results: i) pan-genome analysis of 110 *E. coli* strains (including 53 isolates from IBD studies) revealed discriminating metabolic genes between B2 strains and other strains; ii) both comparative genomic analysis and GEMs suggested that B2 strains have an advantage in degrading and utilizing sugars derived from mucus glycan, and iii) B2 strains display distinct metabolic features, such as their inability to catabolize fructoselysine and psicoselysine, but instead are able to utilize the derivatives produced by neighboring bacteria.

Pan-genome analysis of metabolic genes in 110 strains revealed that B2 strains have distinct metabolic genes. We identified genes that are unique to or more prevalent in B2 strains, including an extra copy of *sucABCD* variant that encodes two important enzymes in the TCA cycle. The importance and function of identified genes need to be experimentally characterized in future studies.

To evaluate the metabolic capabilities of these 110 strains on a systems level, we constructed draft models of 110 strains and examined their *in silico* growth capabilities. B2 strains showed differentiating growth capabilities on certain substrates (Table 1), including amadori products fructoselysine and psicoselysine, potentially because they are able to utilize a derivative of amadori products - butyrate, produced by their neighbouring bacteria.

Both pan-genome and GEM analysis showed that B2 strains have potential advantages that allow them to reside within the human intestinal mucosa. In addition to existing TBP aldolases, GatY and KbaY, that are involved in degrading mucus glycan component, B2 strains contain four extra variants of TBP aldolases, suggesting a potential advantage in utilizing intestinal mucus. Growth simulation with GEMs also suggested that all B2 strains are able to utilize all tested monosaccharides derived from mucus glycan, while 35% of other strains failed to do so.

Although we were able to identify common features among B2 strains, we could not further differentiate subgroups within B2 strains (e.g. AIEC strains versus non-AIEC strains, IBD isolates versus non-IBD isolates). To separate subgroups of B2 strains, we explored diverse datasets (e.g. a growth capability matrix and reaction content matrix generated from GEMs, gene presence/absence matrix generated from pan-genome) using various methods including feature selection method, supervised and unsupervised clustering methods. Such attempts were not entirely successful due to the following reasons: 1. AIEC strains were shown to be a heterogeneous pathotype that displays different genotypes, as shown in previous studies [6]. Therefore, classification of AIEC strains based solely on genomic information remains challenging. 2. Other factors that affect IBD disease state were not taken into account in this analysis, including environmental conditions, host genetics and other microbial community members. A broader approach that takes these factors into consideration could provide valuable insight to the role of *E. coli* strains in IBD patients. 3. Our study utilized only genome sequences of *E. coli* strains, which only delineates the genetic potentials, but not functional states of these strains. Gene expression levels are unavailable for these strains, making it difficult for us to differentiate subgroups of B2 strains (e.g. isolates from healthy controls versus IBD patients). However, we hypothesize that the genes identified here that are unique to B2 strains may be upregulated and used during active IBD. This hypothesis remains to be tested, however. Thus, while we did not observe differences in genetic potential between subgroups of B2 strains, gene expression data would likely help differentiate IBD patient isolates from healthy control isolates based on the different functional states they are in.

## Conclusion

Taken together, these results suggest that the metabolic capabilities of B2 strains vary from those of other strains, enabling them to colonize intestinal mucosa. The results from this study motivate a broad experimental assessment of the ability of B2 *E. coli* strains to utilize different substrates, and further investigations in if they confer growth rate advantages under simulated intestinal conditions. If these strain-specific growth advantages are confirmed in vitro, the nutritional effects on *E. coli* B2 pathophysiology in IBD patients should be rigorously evaluated.

## Methods

### Bacterial genome sequences

We collected 76 genome sequences (including 39 AIEC strains) from various publications [6, 10, 11, 37–40]. We recorded their associated metadata: IBD status of originating patient, anatomic site of collection, serotype, phylotype, and other relevant information where available (see Additional file 1: Table S1). For comparison, we utilized genome sequences of 57 other *E. coli* strains that span various pathotypes as well as commensal strains, most of which are collected from a previous multi-strain *E. coli* study [17]. The quality of the genome sequences varied since they originated from multiple publications. Therefore, we calculated N50 scores of each genome sequence, and only performed analysis on 110 *E. coli* strains (including 53 IBD-associated strains) that have a N50 score greater than 200,000.

### Pan-genome construction and analysis

We first annotated 110 *E. coli* genome sequences and aligned them against each other using CD-HIT [19] with the cutoff for “align average” set to 80%, so that genes with 80% or more sequence similarity are grouped together. We utilized the PATRIC database [41] to extract our sequences and gene calls. All annotations in this resource are called using the same pipeline that consists of assembly with SPADES [42] and annotation with RAST [43]. RAST annotation has also provided EC numbers that allow us to identify metabolic genes. With the alignment, we created a binary matrix that describes the presence or absence of each gene in the strains. We extracted only metabolic genes with enzyme commision numbers. We then performed feature selection using SelectKBest function from the scikit-learn package [20] to select the top features that differentiate B2 and non-B2 strains.

### Analysis of genes involved in mucus degradation

Genes that are involved in degrading the five monosaccharides derived from mucus were primarily identified from a previous study by Ravcheev and Thiele [24]. Gene sequences of the identified genes were collected from the supplementary file of the aforementioned paper. Additional genes involved in galactose metabolism were identified and added based on gene annotation and known pathways. Genome sequences of 110 strains were blasted against 48 identified genes with a threshold of 80% sequence similarity using BLAST [44].

### Protein structural analysis of TBP aldolase

To inspect the possible functions of the additional four predicted class II TBP aldolases in B2 strains, we carried out a comparative analysis of each enzyme’s predicted protein structure. Homology models were obtained from two modeling pipelines (SWISS-MODEL [45] and I-TASSER [46]) in order to compare results from different modeling approaches. Models were compared to the only crystallized structure of TBP aldolase (PDB ID: 1GVF [25]) and a structure of FBP aldolase (PDB ID: 1B57 [26]), which are both bound to a substrate analog of the natural substrate of TBP as well as the cations required for catalysis. Important residues for catalysis were gathered from Hall et al. [25] for comparison in all models. The two sets of homology models were found to be very similar in overall structure and location of these important residues, and as a result the reported results do not differ between the generated models. For visualization, VMD [47] was used along with the MultiSeq plugin [48] to structurally superimpose all models.

### Draft model reconstruction of other *E. coli* strains

We first created an *E. coli* pan model that combines all the genes, reactions, and metabolites in the 55 *E. coli* models reconstructed by Monk et al. [17]. In addition, in order to incorporate any novel metabolic functions in the 110 strains that are absent from the previously-built *E. coli* models, we identified 340 metabolic genes in the constructed pan-genome that are absent from the previously studied 55 strains. However, the majority of the 340 genes are variants of existing genes, and only 96 genes may encode new functions. For these 96 genes, we utilized Uniprot [49] database to identify associated reactions, with the following criteria to select the reactions to include: 1) Not involved in DNA/RNA modification, as suggested by the established GEM reconstruction protocol [50]; 2) experimentally proven to be present in *E. coli*; 3) have a defined reaction with specificity; 4) do not duplicate with existing reactions in the 55 GEMs. In the end, we only identified five new metabolic reactions that fulfill all above requirements (see Additional file 1: Table S3), mainly because these strains are not as well studied compared to the previous 55 strains, and little experimental evidence was found for the majority of the investigated metabolic functions. We then added these new reactions to the pan model created from the previous 55 models. To create strain-specific draft models, we mapped the 110 *E. coli* genome sequences to all the genes in the pan model using BLAST [44], and set a homology threshold of 80% for a gene to be considered present in the strain. The missing genes and their correlated reactions and metabolites in each strain were removed from the pan model to create strain-specific draft models.

### *In silico* growth simulations

Growth simulation for *E. coli* draft models were performed using COBRApy [51]. We used M9 minimal media with the lower bound of exchange reactions for the following substrate set to -1000: *Ca*^2+^, *Cl*^−^, *CO*^2^, *Co*^2+^, *Cu*^2+^, *Fe*^2+^, *Fe*^3+^, *H*^+^, *H*^2^O, *K*^+^, *Mg*^2+^, *Mn*^2+^, *MoO*^4 2^, *Na*^+^, *Ni*^2+^, *SeO*_4_^2^−, *SeO*_3_^2^+, and *Zn*^2+^. Another essential substrate is cob(I)alamin, for which the exchange reaction has a lower bound of -0.01. In addition, the default carbon source is glucose with default lower bound set to be -20, while the default nitrogen, sulfur and phosphate sources are *NH*_4_^−^, *SO*_4_^2^, *HPO*_4_^2^ with the lower bounds all set to be -1000. We evaluated if sole carbon, nitrogen, sulfur or phosphate substrates supported growth by setting the lower bound of the exchange reaction of the default substrate to 0, and added sole substrates by setting the lower bound of exchange reaction to -10. We simulated growth under aerobic conditions with the lower bound of the oxygen exchange reaction set to -20. If the simulated growth rate is greater than 1% of the original growth rate (when all default nutrient sources are used), the strain is considered to be able to grow under the tested condition.

Among all 110 strains tested, three draft GEMs were not able to simulate growth on the majority of the substrates, potentially due to auxotrophy: *E. coli* str K-12 substr DH10B, *E. coli* O111 H-str 11128, *E. coli* NA114. These strains were therefore excluded from the following growth capability analysis.

We used SelectKBest function in scikit-learn package [20] to select the top 10 growth substrates that differentiate B2 and non-B2 strains, with the score function set to “f_classif”. We then summarized the percentage strains in each pathotype that could utilize these substrates in Table 1. Note that in Table 1 we classified pathotypes to B2 group and non-B2 group, but with a few exceptions in both groups: i.e. non-B2 strains in the ExPEC group and B2 strains in the commensal group.

## Additional files

Additional file 1 **Table S1:** Metadata of 110 strains. **Table S2:** Genes associated with mucus degradation. **Table S3:** Metabolic reactions added to previous reconstruction. (XLSX 32 kb)

Additional file 2 Additional analysis and results. (PDF 1776 kb)

## Abbreviations

ABC: ATP-binding cassette
AIEC: Adherent-invasive *E. coli*
EC: Enzyme Commission
ExPEC: Extra-intestinal *E. coli*
FBP: Fructose-1,6-bisphosphate
FDR: False Discovery Rate
GalNAc: N-acetylgalactosamine
GEM: Genome-scale model
GlcNAc: N-acetylglucosamine
IBD: Inflammatory bowel disease
InPEC: Intra-intestinal *E. coli*
LPS: Lipopolysaccharides
Neu5Ac: N-acetylneuraminic acid
TBP: Tagatose 1,6-bisphosphate
TCA: Tricarboxylic acid
