# Supplementary material for: Escherichia coli B2 strains prevalent in inflammatory bowel disease patients have distinct metabolic capabilities that enable colonization of intestinal mucosa
Source: BMC Syst Biol. 2018 Jun 11;12:66. doi: 10.1186/s12918-018-0587-5 (PMC5996543; doi:10.1186/s12918-018-0587-5)
Supplement: Supplementary file 2 — Additional analysis and results. (PDF 1776 kb) [file 12918_2018_587_MOESM2_ESM.pdf]

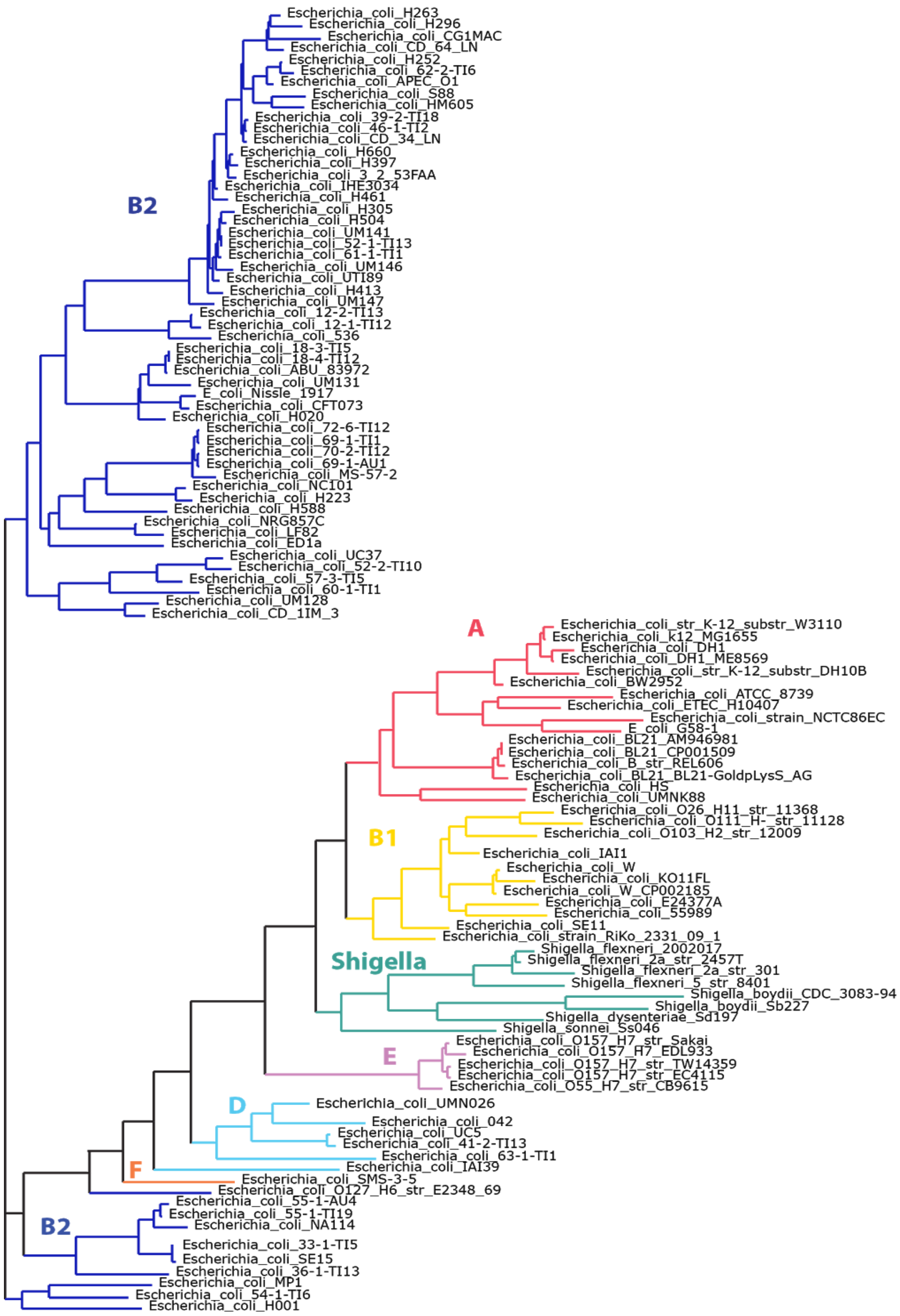

Figure S1: Phylogenetic tree of 110 strains.

E.coli has an open pan-genome

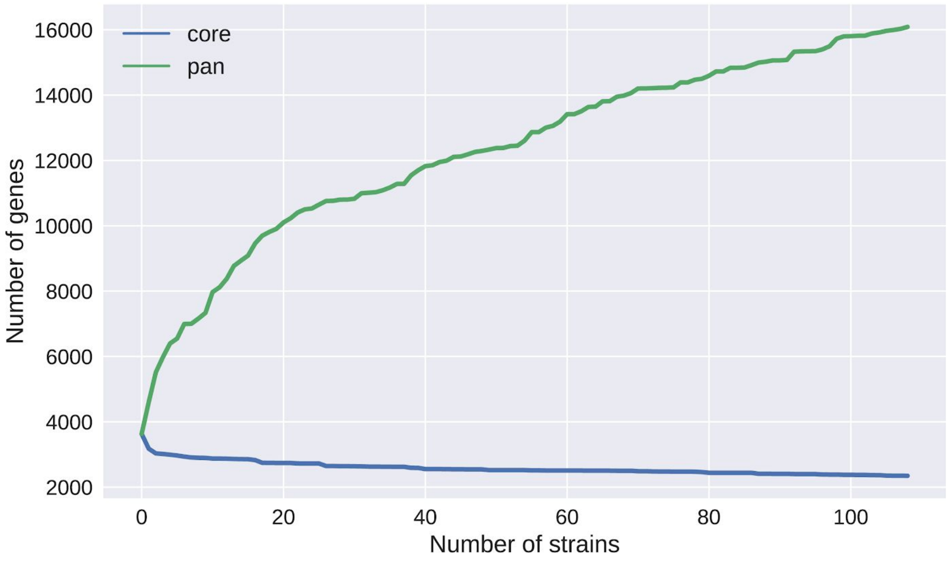

Figure S2: Number of pan and core genes for the studied 110 *E.coli* strains

We observed that the number of pan gene increased for every additional strains added, implying that *E.coli* has an open pan-genome. Additionally, previous studies have also shown that *E.coli* has an open pan-genome [1,2].

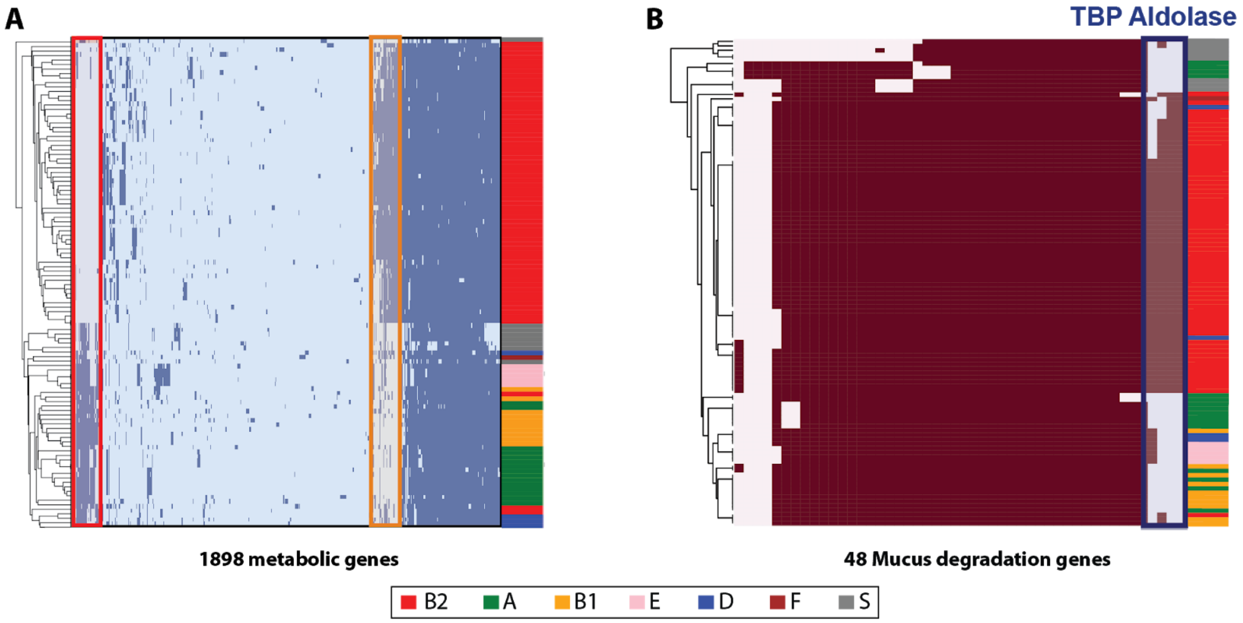

Figure S3: Figure 1A and Figure 1B with all phylogroups labeled by color. B2 strains mostly clustered together and shared similar features , with only a few exceptions in both figures.

Visualization and detailed structural analysis of identified TBP aldolases

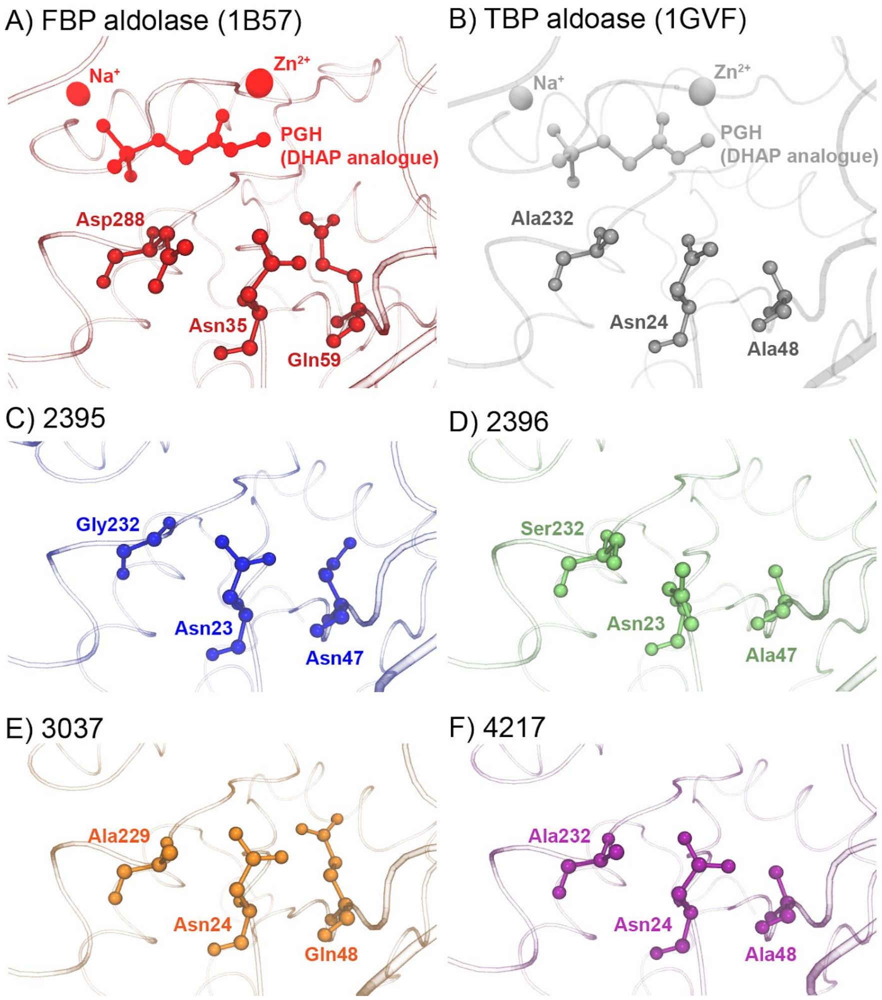

Figure S4: Visualization of important residues in known TBP aldolase, FBP aldolase and four identified proteins that were annotated as TBP aldolases.

The four predicted TBP aldolases were found to be structurally more similar to the crystallized TBP aldolase. One differentiating feature between TBP and FBP aldolases is the extended sequence of amino acids in FBP aldolase that creates the known ɑ10 loop-ɑ11 arm [3], which is absent in TBP aldolase or the tested unknown proteins (Table S4). Moreover, we also observed differences in the substrate binding sites between TBP aldolase and FBP aldolase. It was hypothesized these variations in residues result in the different extent of steric restrictions in these two enzymes: FBP aldolase is highly specific for FBP, while TBP aldolase has poor stereochemical control and a lower substrate specificity. Each predicted TBP aldolase contains a different set of residues

which have the potential to greatly alter these steric restrictions and allow a wider range of substrates to enter the binding site (Table S4 and Figure S4).

Table S4: Presence or absence of important protein features that differentiate TBP and FBP aldolases in all six investigated enzymes

	**PDB ID or template used for modeling**	**α10 loop - α11 arm**	**G3P substrate binding residues**		
**FBP aldolase**	1B57	x	Asn35	Gln59	Asp288
**TBP aldolase**	1GVF		Asn24	Ala48	Ala232
**fig|749528.3.peg.3037**	3C4U (template)		Asn24	Gln48	Ala229
**fig|749528.3.peg.4217**	1RVG (template)		Asn24	Ala48	Ala232
**fig|749528.3.peg.2935**	3Q94 (template)		Asn23	Asn47	Gly232
**fig|749528.3.peg.2936**	5U4N (template)		Asn23	Ala47	Ser232

Copy number variation of core metabolic genes

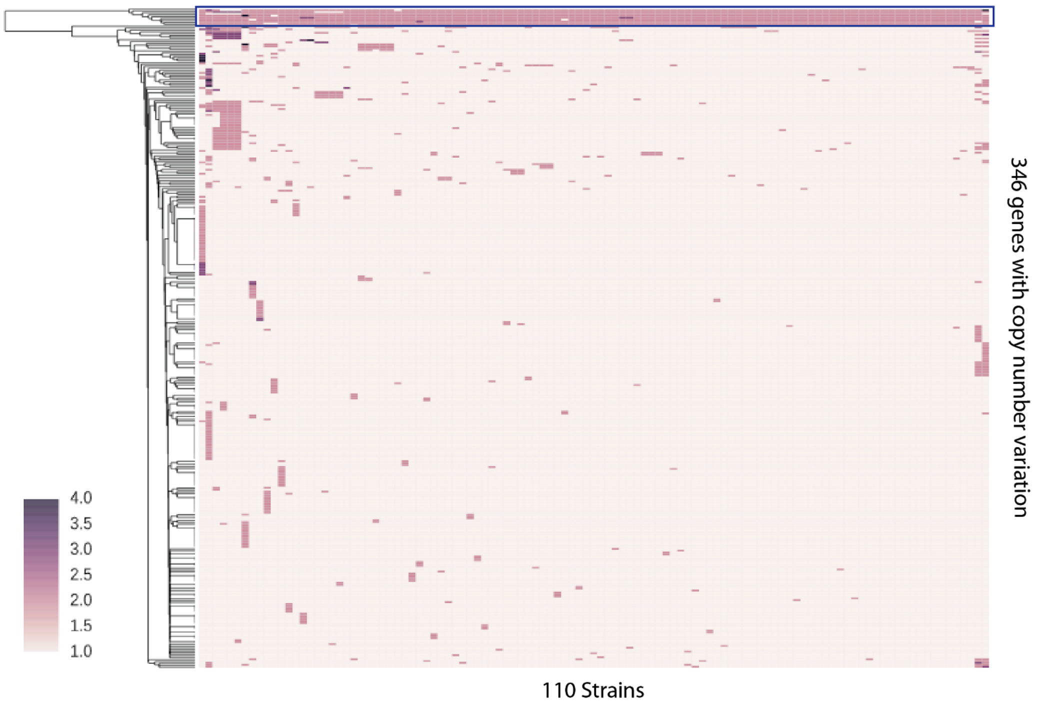

Figure S5: Copy number for 346 genes with copy number variation in all 110 strains.

We investigated the copy number variation for 1081 core metabolic genes in 110 strains. Only 346 genes have copy number variation (>1 copy), and the rest of the genes only have one copy across all strains. Among the 346 genes, most genes only have one copy in the majority of the strains (Fig. S5), while 9 genes have a copy number of 2 in most strains (highlighted by blue box in Fig. S5). We also calculated the number of genes with more than one copy each strain (Fig. S6). We did not observe any general pattern, since for most genes, copy number only varies for a few strains. We found that *Shigella* strains tend to have copy number variation for more genes ranging from 40 genes to 75 genes, while most other *E. coli* strains have copy number variation for less than 20 genes. The only other interesting observations is that one *E. coli* strain - *E.coli* Nissle 1917 has copy number variation for 67 genes. We further investigated the genes which Nissle 1917 have more copies of, and showed that they are enriched for various functions including methionine metabolic process (FDR adjusted p-value = 4.74e-2), ATP hydrolysis coupled cation transmembrane transport (FDR adjusted p-value = 1.16e-2), sulfur compound biosynthetic processes (FDR adjusted p-value = 4.83e-2), tRNA metabolic processes (FDR adjusted p-value = 3.9e-2), RNA modification (FDR adjusted p-value = 3.87e-2). More research needs to be done on genome assembly quality of *E.coli* Nissle 1917 to further explain the above observation.

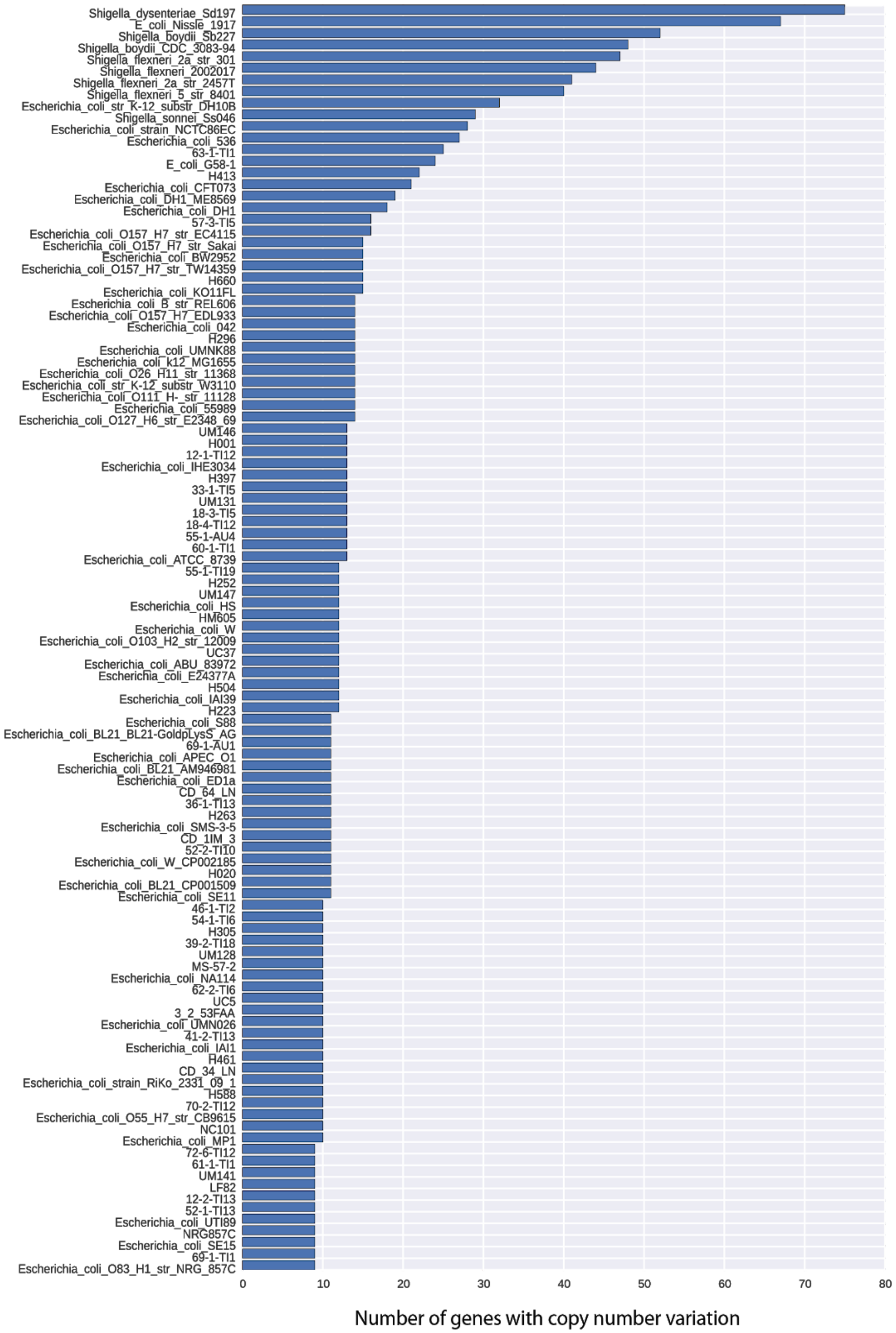

Figure S6: Number of genes with copy number variation for each strain.

References

1. Rasko DA, Rosovitz MJ, Myers GSA, Mongodin EF, Fricke WF, Gajer P, et al. The pangenome structure of Escherichia coli: comparative genomic analysis of E. coli commensal and pathogenic isolates. J Bacteriol. 2008;190:6881–93.

2. Gordienko EN, Kazanov MD, Gelfand MS. Evolution of pan-genomes of Escherichia coli, Shigella spp., and Salmonella enterica. J Bacteriol. 2013;195:2786–92.

3. Hall DR, Bond CS, Leonard GA, Watt CI, Berry A, Hunter WN. Structure of tagatose-1,6-bisphosphate aldolase. Insight into chiral discrimination, mechanism, and specificity of class II aldolases. J Biol Chem. 2002;277:22018–24.
